# Epithelial miR-206 targets CD39/extracellular ATP to upregulate airway IL-25 and TSLP in type 2–high asthma

**DOI:** 10.1172/jci.insight.148103

**Published:** 2021-06-08

**Authors:** Kan Zhang, Yuchen Feng, Yuxia Liang, Wenliang Wu, Chenli Chang, Dian Chen, Shengchong Chen, Jiali Gao, Gongqi Chen, Lingling Yi, Dan Cheng, Guohua Zhen

**Affiliations:** 1 Division of Pulmonary and Critical Care Medicine, Department of Internal Medicine, Tongji Hospital, Tongji Medical College, Huazhong University of Science and Technology, Wuhan, China.; 2 Key Laboratory of Respiratory Diseases, National Health Commission of People’s Republic of China, and National Clinical Research Center for Respiratory Diseases, Wuhan, China.; 3 Department of Respiratory and Critical Care Medicine, The Affiliated Suzhou Hospital of Nanjing Medical University, Suzhou, China.; 4 Department of Respiratory and Critical Care Medicine, Renmin Hospital of Wuhan University, Wuhan, China.

**Keywords:** Pulmonology, Asthma

## Abstract

The epithelial cell–derived cytokines IL-25, IL-33, and thymic stromal lymphopoietin (TSLP) initiate type 2 inflammation in allergic diseases, including asthma. However, the signaling pathway regulating these cytokines expression remains elusive. Since microRNAs are pivotal regulators of gene expression, we profiled microRNA expression in bronchial epithelial brushings from type 2–low and type 2–high asthma patients. miR-206 was the most highly expressed epithelial microRNA in type 2–high asthma relative to type 2–low asthma but was downregulated in both subsets compared with healthy controls. CD39, an ectonucleotidase degrading ATP, was a target of miR-206 and upregulated in asthma. Allergen-induced acute extracellular ATP accumulation led to miR-206 downregulation and CD39 upregulation in human bronchial epithelial cells, forming a feedback loop to eliminate excessive ATP. Airway ATP levels were markedly elevated and strongly correlated with IL-25 and TSLP expression in asthma patients. Intriguingly, airway miR-206 antagonism increased Cd39 expression; reduced ATP accumulation; suppressed IL-25, IL-33, and Tslp expression and group 2 innate lymphoid cell expansion; and alleviated type 2 inflammation in a mouse model of allergic airway inflammation. In contrast, airway miR-206 overexpression had opposite effects. Overall, epithelial miR-206 upregulates airway IL-25 and TSLP expression by targeting the CD39–extracellular ATP axis, which represents a potentially novel therapeutic target in type 2–high asthma.

## Graphical abstract

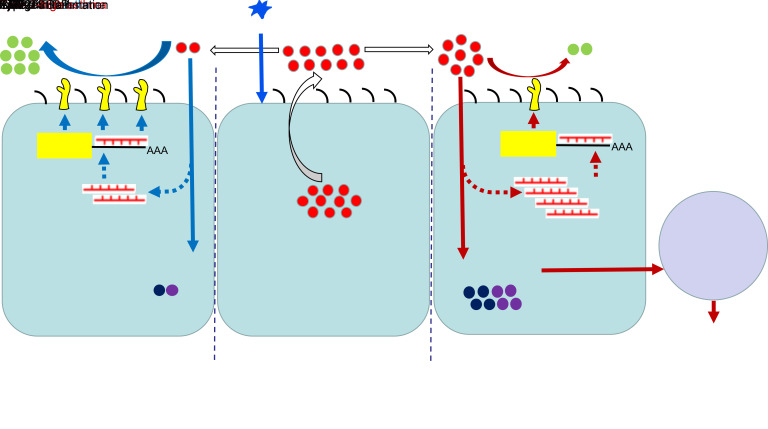

## Introduction

Asthma is a heterogeneous disease with different phenotypes and endotypes and with variable responses to management approaches (1, 2). A prominent endotype of asthma is the presence of type 2 inflammation (3, 4). Airway epithelial cells play pivotal roles in the initiation of type 2 inflammation by expressing IL-25, IL-33, and thymic stromal lymphopoietin (TSLP) (5–7). These epithelial cell–derived cytokines act on innate immune cells, including DCs, group 2 innate lymphoid cells (ILC2s), and mast cells (8–15). Recent identification of memory Th2 cells with high expression of receptors for IL-25, IL-33, and TSLP supports a role of these cytokines in adaptive immune responses in allergy (16, 17). IL-25 (18, 19), IL-33 (20), and TSLP (11) have each been reported to be indispensable and sufficient for type 2 cytokine production, eosinophilic airway inflammation, and AHR in certain mouse models. In human asthma, various expression patterns of these cytokines have been reported (21–25). Although IL-25, IL-33, and TSLP are critical in type 2 airway inflammation, the upstream signaling pathway regulating their expression remains elusive.

When exposed to environmental stimuli, airway epithelial cells rapidly express danger signals, such as adenosine triphosphate (ATP) and uric acid, to alert the immune system (26, 27). Allergen exposure enhanced airway ATP concentrations in human asthma and sensitized mice. Neutralizing airway ATP or blocking purine signaling suppressed airway inflammation in allergen-sensitized and challenged mice (28). The extracellular ATP concentration is tightly controlled by ectonucleoside triphosphate diphosphohydrolases (ENTPDs) (29). CD39 (encoded by *ENTPD1*) catalyzes the degradation of extracellular ATP and ADP, and it is expressed in airway epithelial cells (30). Inhibition of Cd39 activity increased the bronchoalveolar lavage fluid (BALF) ATP concentration and intensified OVA-induced bronchospasm in mice (31). *Cd39*-deficient mice exhibited worsened airway inflammation and mucus overproduction after allergen sensitization and challenge (32), whereas paradoxically alleviated airway inflammation was also reported in *Cd39*-deficient mice (33). Recently, it was reported that ATP serves as a sensor for an airborne allergen to trigger IL-33 release in airway epithelial cells (34). Together, these studies suggest that the CD39–extracellular ATP axis may regulate IL-25, IL-33, and TSLP in asthma.

miRNAs regulate gene expression by promoting the catabolism of the target transcripts, as well as attenuating their translation. A growing body of evidence indicates that miRNAs play important roles in epithelial cell, ILC2, and Th2 cell differentiation and function in asthma (35–38). miR-19a promotes IL-5 and IL-13 expression in ILC2s and Th2 cells (37, 38). Let-7 miRNA regulates IL-13 expression and allergic airway inflammation (39, 40). Global miRNA expression in airway epithelial cells is altered in asthma (36, 41). However, the difference in epithelial miRNA expression between type 2–low and type 2–high asthma remains unknown. We hypothesized that the differentially expressed epithelial miRNAs between the 2 asthma subsets may contribute to IL-25, IL-33, and TSLP expression in asthma.

In this study, we profiled the epithelial miRNA expression in type 2–low and type 2–high asthma patients. We found that miR-206, the most highly expressed miRNA in type 2–high asthma relative to type 2–low asthma, targets the CD39–extracelluar ATP axis to regulate IL-25 and TSLP expression in cultured bronchial epithelial cells. Airway ATP levels were increased and strongly correlated with elevated IL-25 and TSLP expression in type 2–high asthma patients. In a mouse model of asthma, manipulation of airway miR-206 expression altered IL-25, IL-33, and Tslp expression; ILC2 expansion; and type 2 airway inflammation.

## Results

### Differentially expressed epithelial miRNAs, including miR-206, between type 2–low and type 2–high asthma.

**Figure 1:**
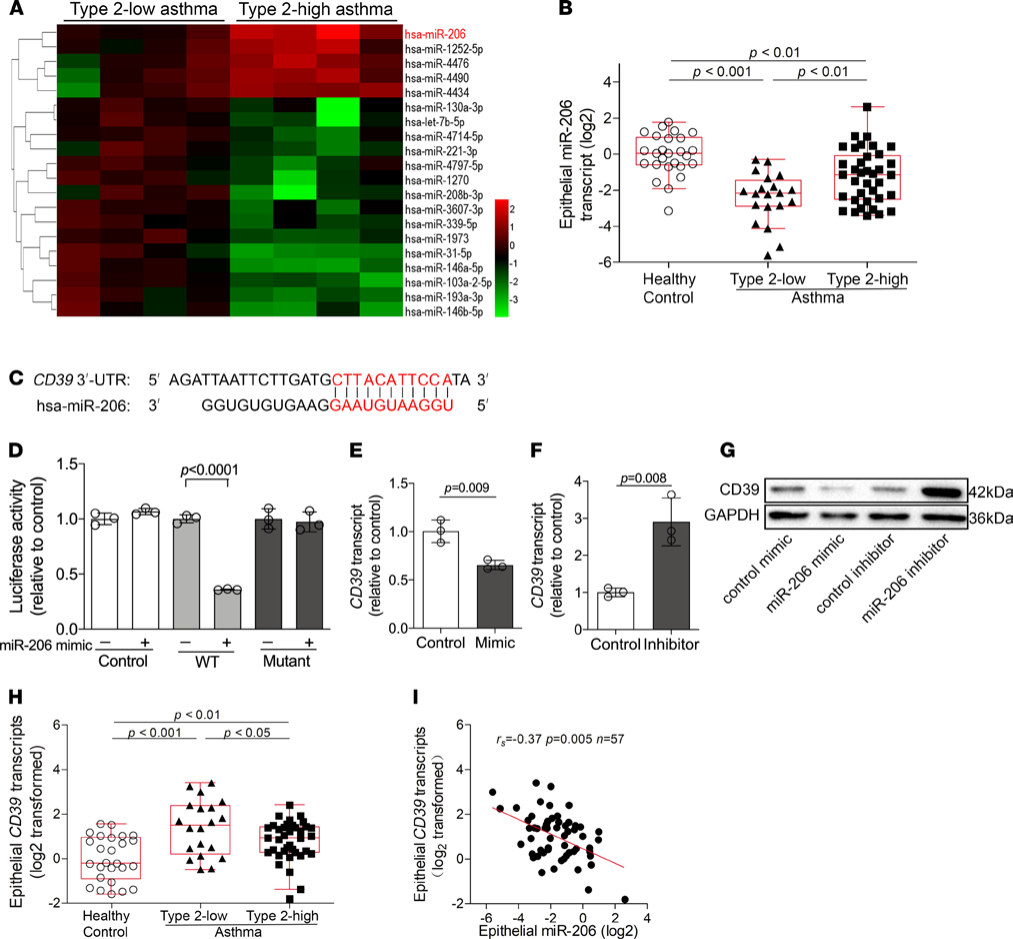
An epithelial miRNA differentially expressed between type 2–low and type 2–high asthma, miR-206, targets CD39. (**A**) Twenty differentially expressed epithelial miRNAs between subjects with type 2–low asthma (*n* = 4) and type 2–high asthma (*n* = 4) were identified using microarrays. Each column represents data from 1 subject. Colors represent fold change relative to the mean value of type 2–low asthma. (**B**) miR-206 transcript levels in bronchial brushings from healthy control (*n* = 26), type 2–low (*n* = 20), and type 2–high asthma patients (*n* = 37) were determined by qPCR. The transcript levels are relative to the median of healthy controls and log_2_ transformed. Data represent medians with IQRs (1-way ANOVA with Bonferroni’s post hoc test). (**C**) The 3′-UTR of *CD39* contains a region that matches the seed sequence of hsa–miR-206. (**D**) 3′-UTR luciferase reporter assay with vector harboring WT, mutant *CD39* 3′-UTR, or no 3′-UTR (control) cotransfected with miR-206 mimic or nontargeting control. Luciferase activity was measured with a dual-luciferase reporter assay system. The firefly luciferase activity was normalized to Renilla luciferase activity. *n* = 3 per group. (**E** and **F**) *CD39* transcript levels in BEAS-2B cells after transfection with miR-206 mimic (**E**) or inhibitor (**F**) were determined by qPCR. The transcript levels are relative to the mean value of control group (2-tailed Student’s *t* test). *n* = 3 per group. The data are represented as mean ± SD. (**G**) CD39 protein expression in BEAS-2B cells after transfection with miR-206 mimic and inhibitor was determined by Western blotting. (**H**) *CD39* transcript levels in bronchial brushings from healthy control (*n* = 26), type 2–low asthma (*n* = 20), and type 2–high asthma patients (*n* = 37) were determined by qPCR. Data are expressed and compared as in **B**. (**I**) Spearman’s rank order correlation assay between epithelial *CD39* and miR-206 transcript levels in all asthma patients (*n* = 57).

We profiled miRNA expression in bronchial epithelial brushings from type 2–low asthmatics (*n* = 4) and type 2–high asthmatics (*n* = 4) using a miRNA microarray. The type 2 status of asthma was defined by the expression of the type 2 signature genes (*CLCA1*, *POSTN*, and *SERPINB10*) in the epithelial brushings as previously reported (3, 42). We found that 20 miRNAs were significantly differentially expressed between the 2 subsets of asthma (Figure 1A). The data are available at GEO (http://www.ncbi.nlm.nih.gov/geo/, accession no. GSE142237). Of note, miR-206 was the most highly expressed miRNA in type 2–high asthma relative to type 2–low asthma. Several other differentially expressed miRNAs, including miR–31-5p, miR–146a-5p, miR–146b-5p, and miR–221-3p, have been implicated in asthma pathogenesis (43–45). Epithelial miR–221-3p expression was shown to be associated with airway eosinophilia and the expression of type 2 signature genes in asthma patients in our previous study (46).

We next examined the expression of miR-206 in a cohort including type 2–low asthmatics (*n* = 20), type 2–high asthmatics (*n* = 37), and healthy controls (*n* = 26) using quantitative PCR (qPCR). Compared with type 2–low asthmatics, type 2–high asthmatics had lower methacholine PD_20_ (i.e., provocative dosage required to cause a 20% decline in forced expiratory volume in the first second [FEV_1_]), higher eosinophil counts in induced sputum and blood, and higher fractional exhaled nitric oxide (FeNO) and serum IgE levels (Table 1). Consistent with the microarray data, epithelial miR-206 expression was higher in type 2–high asthma than in type 2–low asthma. However, compared with healthy controls, epithelial miR-206 transcript levels were decreased in both type 2–low and type 2–high asthma (Figure 1B). Our data suggest that epithelial miR-206 expression is downregulated in asthma and associates with airway type 2 inflammation.

### The expression of CD39, a target of miR-206, is upregulated in airway epithelial cells in asthma.

We predicted candidate target genes of miR-206 by using online algorithms (DIANA-microT, miRanda, miRWalk, PicTar, and TargetScan). CD39 (encoded by *ENTPD1*), the ectoenzyme catalyzing the degradation of extracellular ATP and ADP, was one of the candidate targets. The 3′-untranslated region (UTR) of *CD39* contains nucleotides matching the seed sequence of *hsa–miR-206* (Figure 1C). Transfection with the miR-206 mimic decreased the luciferase activity when cotransfected with the vector harboring the WT *CD39* 3′-UTR but had no effect on the luciferase activity when cotransfected with the vector containing the mutant 3′-UTR or empty vector (Figure 1D). This indicates that miR-206 may directly act on the 3′-UTR of *CD39* mRNA. Furthermore, inhibition of miR-206 expression enhanced CD39 mRNA and protein expression, whereas overexpression of miR-206 suppressed CD39 expression (Figure 1, E–G). These data indicate that *CD39* is a target of miR-206.

In human asthma, we found that *CD39* transcript levels were significantly increased in bronchial brushings from type 2–low and –high asthma patients compared with controls. Moreover, epithelial *CD39* expression was lower in type 2–high asthma than in type 2–low asthma (Figure 1H). In support of *CD39* as a target of miR-206, epithelial *CD39* transcript levels negatively correlated with epithelial miR-206 expression in asthma patients (Figure 1I).

### Extracellular ATP accumulation induces miR-206 downregulation and CD39 upregulation in human bronchial epithelial cells.

**Figure 2:**
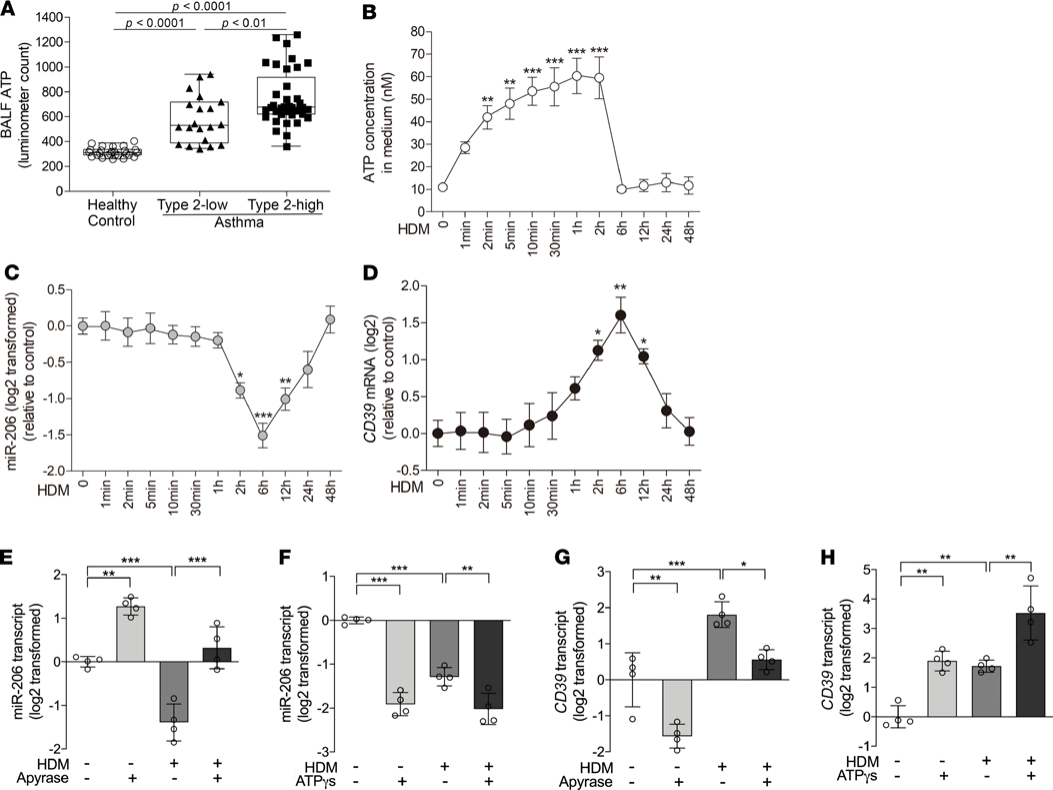
Acute extracellular ATP accumulation is responsible for allergen-induced miR-206 downregulation and CD39 upregulation in bronchial epithelial cells. (**A**) ATP levels in BALF from healthy control (*n* = 26), type 2–low (*n* = 20), and type 2–high asthma patients (*n* = 37) were determined by luciferase bioluminescence. Data are expressed as median values withIQRs. The lines within the boxes represent medians, and the bounds of the boxes represent IQRs. The whiskers are plotted using Tukey method. One-way ANOVA with Bonferroni’s post hoc test was performed. (**B**) ATP concentration in culture medium collected at indicated time points after HDM stimulation was measured using luciferase bioluminescence. (**C** and **D**) Transcript levels of miR-206 (**C**) and *CD39* (**D**) in HBE cells harvested at the indicated time points after HDM stimulation were determined by qPCR. *n* = 4 wells per time point combined from 2 experiments using HBE cells from 2 healthy donors. The data are represented as mean ± SD. *P < 0.05; **P < 0.01; ***P < 0.001 (1-way ANOVA with Bonferroni’s post hoc test). (**E** and **F**) miR-206 transcript levels in HBE cells pretreated with apyrase or saline for 2 hours before addition of HDM and stimulation for 6 hours (**E**), and treated with ATPγS or saline with or without HDM for 6 hours (**F**). (**G** and **H**) *CD39* transcript levels in HBE cells pretreated with apyrase or saline for 2 hours before addition HDM and stimulation for 6 hours (**G**), and treated with ATPγS or saline with or without HDM for 6 hours (**H**). The transcript levels are relative to the mean value of control group and log_2_ transformed. *n* = 4 wells per group combined from 2 experiments using HBE cells from 2 healthy donors. The data are represented as mean ± SD. *P < 0.05; **P < 0.01; ***P < 0.001 (1-way ANOVA with Bonferroni’s post hoc test).

It was reported that BALF ATP concentration was increased in asthma patients after allergen provocation (28). In BALF from our symptomatic and treatment-naive asthma patients, we measured ATP using luciferase bioluminescence. BALF ATP levels were markedly increased in both type 2–low and type 2–high asthma patients compared with controls (Figure 2A). Moreover, BALF ATP levels were higher in type 2–high asthma than in type 2–low asthma. This suggests that extracellular ATP accumulates in the airways of symptomatic asthma patients.

Extracellular ATP accumulation upregulates CD39 expression in airway epithelial cells, and upregulated CD39 catalyzes degradation of excessive extracellular ATP to maintain the homeostasis in the microenvironment (47). To determine whether the miR-206–CD39 axis responds to extracellular ATP accumulation, we performed air-liquid interface culture of human bronchial epithelial (HBE) cells from healthy donors. House dust mite (HDM), the most clinically relevant allergen, rapidly increased ATP concentrations in culture medium within 2 minutes. The ATP concentration peaked at 1–2 hours and declined to baseline by 6 hours (Figure 2B). HDM exposure decreased miR-206 expression from 2 hours to 12 hours, with maximum inhibition at 6 hours (Figure 2C), and increased the *CD39* mRNA level from 2 hours to 12 hours, peaking at 6 hours (Figure 2D). Furthermore, elimination of extracellular ATP by pretreatment with apyrase suppressed HDM-induced miR-206 downregulation and CD39 upregulation at 6 hours in HBE cells (Figure 2, E and G). The exogenous ATP analog ATPγS directly decreased miR-206 expression, increased CD39 expression, and intensified HDM-induced miR-206 downregulation and CD39 upregulation (Figure 2, F and H). Similarly, we found that *Alternaria*-induced extracellular ATP accumulation was also required for miR-206 downregulation and CD39 upregulation in BEAS-2B cells (Supplemental Figure 1; supplemental material available online with this article; https://doi.org/10.1172/jci.insight.148103DS1). Together, our data suggest that allergen-induced acute accumulation of extracellular ATP downregulates miR-206 and upregulates CD39 expression in airway epithelial cells. This may represent a protective mechanism to eliminate excessive extracellular ATP.

### Higher ATP levels are associated with elevated IL-25 and TSLP expression in type 2–high asthma.

**Figure 3:**
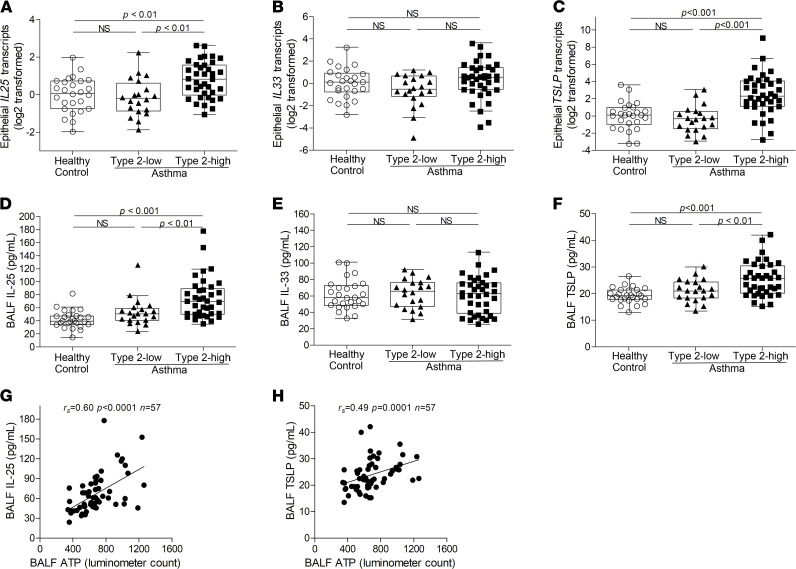
Airway IL-25 and TSLP expression is elevated in type 2–high asthma and correlated with BALF ATP levels. (**A**–**C**) The transcripts of *IL25* (**A**), *IL33* without exons 3 and 4 (**B**), and the long isoform of *TSLP* (**C**) in bronchial epithelial brushings from healthy control (*n* = 26), type 2–low (*n* = 20), and type 2–high asthma patients (*n* = 37) were determined using qPCR with TaqMan primers and probes. For detection of *IL33* transcripts without exons 3 and 4, RNase H–dependent qPCR was performed. The transcript levels are relative to the median value of healthy controls and log_2_ transformed. (**D**–**F**) IL-25 (**D**), IL-33 (**E**), and TSLP (**F**) protein levels in BALF from healthy control (*n* = 26), type 2–low (*n* = 20), and type 2–high asthma patients (*n* = 37) were determined using ELISA. Data are expressed as median values with IQRs. The lines within the boxes represent medians, and the bounds of the boxes represent IQRs. The whiskers are plotted using Tukey method. One-way ANOVA with Bonferroni’s post hoc test was performed. (**G** and **H**) Spearman’s rank order correlation assays between BALF ATP levels and BALF IL-25 protein levels (**G**) and TSLP protein levels (**H**).

Allergens stimulate the release of ATP as an alarmin from airway epithelial cells to induce the expression of IL-33 (34). We next examined airway IL-25, IL-33, and TSLP expression in bronchial epithelial brushings and BALF using qPCR and ELISA, respectively. Epithelial *IL25* transcript levels and BALF IL-25 protein levels were significantly higher in type 2–high asthma patients than in type 2–low asthma patients and controls (Figure 3, A and D). There were multiple splice variants of the *IL33* transcript, and the protein encoded by the *IL33* transcript without exons 3 and 4 was secreted as an active cytokine (48). We examined the expression of *IL33* transcripts without exons 3 and 4 in bronchial epithelial brushings using RNase H–dependent qPCR as previously reported (48). However, there were no significant differences in this *IL33* transcript between the 2 asthma subsets and controls. Additionally, we did not detect a significant difference in IL-33 protein levels in BALF (Figure 3, B and E). TSLP has short and long isoforms, and the long isoform is induced during inflammation (49). We found that the long *TSLP* transcripts and BALF TSLP protein levels were higher in type 2–high asthma patients than in type 2–low asthma patients and control subjects (Figure 3, C and F). Our data suggest that airway expression of IL-25 and TSLP, but not IL-33, is elevated in type 2–high asthma.

Importantly, BALF ATP levels were strongly correlated with BALF IL-25 and TSLP protein levels (Figure 3, G and H). This indicates that the more prominent accumulation of airway ATP may be responsible for the elevated IL-25 and TSLP expression in type 2–high asthma.

### Extracellular ATP is essential for allergen-induced IL-25 and TSLP expression in HBE cells.

**Figure 4:**
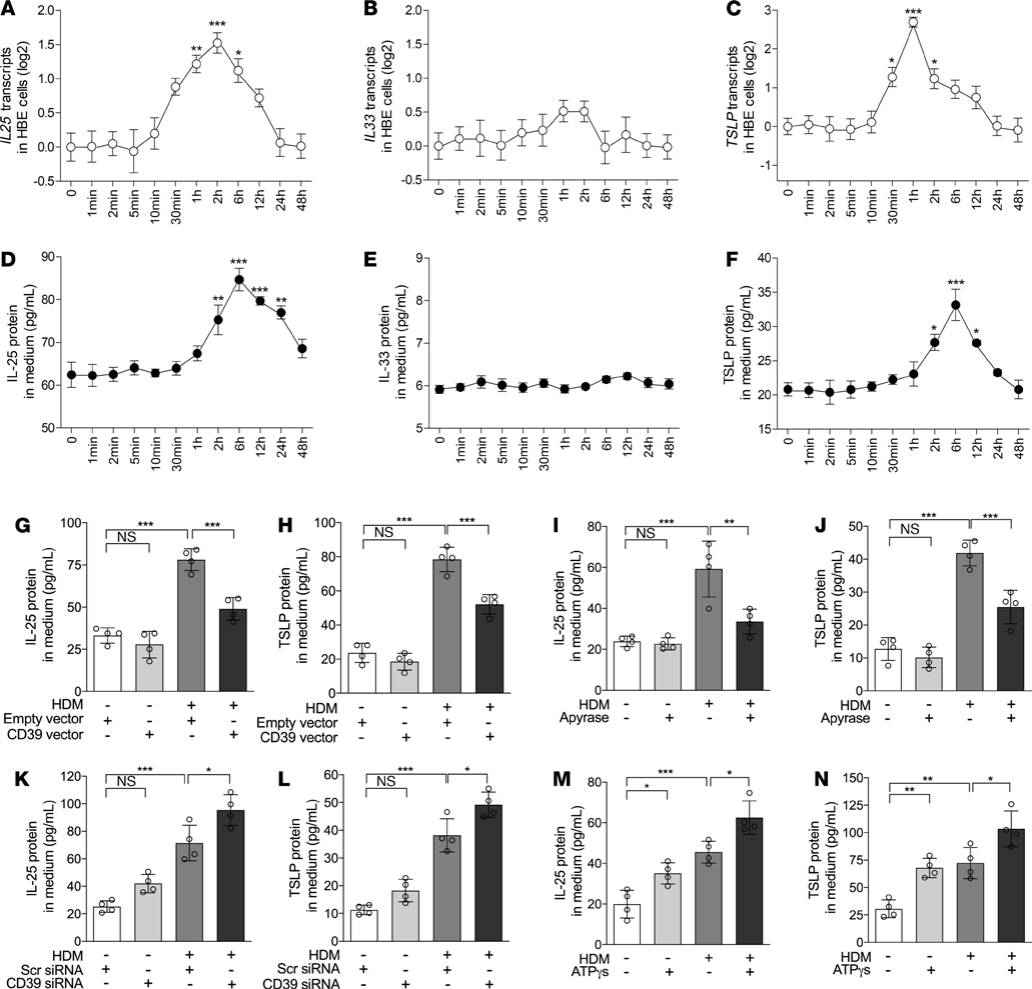
Extracellular ATP is required and sufficient for IL-25 and TSLP expression in bronchial epithelial cells. (**A**–**C**) The transcripts of *IL25* (**A**), *IL33* without exons 3 and 4 (**B**), and the long isoform of *TSLP* (**C**) in HBE cells harvested at the indicated time points after HDM stimulation were determined by qPCR. (**D**–**F**) IL-25 (**D**), IL-33 (**E**), and TSLP (**F**) protein levels in culture medium collected at the indicated time points after HDM stimulation were determined using ELISAs. *n* = 4 wells per time points combined from 2 experiments using HBE cells from 2 healthy donors. Data are mean ± SD. *P < 0.05; **P < 0.01; ***P < 0.001 (1-way ANOVA with Bonferroni’s post hoc test). (**G** and **H**) IL-25 (**G**) and TSLP (**H**) protein levels in culture medium after transfection with empty or CD39 expression vector and stimulation with or without HDM for 6 hours were determined using ELISAs. (**I** and **J**) IL-25 (**I**) and TSLP (**J**) protein levels in culture medium after pretreatment with apyrase or saline and stimulation with or without HDM for 6 hours were determined using ELISAs. (**K** and **L**) IL-25 (**K**) and TSLP (**L**) protein levels in culture medium after transfection with scrambled or CD39 siRNA and stimulation with or without HDM for 6 hours were determined using ELISAs. (**M** and **N**) IL-25 (**M**) and TSLP (**N**) protein levels in culture medium after treatment with ATPγS or saline and with or without HDM for 6 hours were determined using ELISAs. *n* = 4 wells per group combined from 2 experiments using HBE cells from 2 healthy donors. Data are mean ± SD. *P < 0.05; **P < 0.01; ***P < 0.001 (1-way ANOVA with Bonferroni’s post hoc test).

We next examined the role of extracellular ATP in HDM-induced IL-25, IL-33, and TSLP expression in HBE cells cultured on an air-liquid interface. We found that HDM stimulation increased *IL25* mRNA expression in HBE cells and IL-25 protein levels in basal-lateral medium, peaking at 2 hours and 6 hours, respectively (Figure 4, A and D). HDM also increased *TSLP* (the long transcript variant) mRNA and protein expression, which peaked at 1 hour and 6 hours, respectively (Figure 4, C and F). However, HDM stimulation did not alter the expression of *IL33* (the transcript without exons 3 and 4) mRNA or protein (Figure 4, B and E). Elimination of extracellular ATP through CD39 overexpression or the use of apyrase suppressed HDM-induced IL-25 and TSLP protein expression at 6 hours (Figure 4, G–J). In contrast, enhancing extracellular ATP by CD39 knockdown or by adding ATPγS further increased HDM-induced IL-25 and TSLP protein expression (Figure 4, K–N). Similarly, *Alternaria*-induced extracellular ATP accumulation promoted IL-25 and TSLP expression in BEAS-2B cells (Supplemental Figure 2). These data suggest that allergen-induced acute extracellular ATP accumulation is required for IL-25 and TSLP upregulation in airway epithelial cells.

### miR-206 regulates allergen-induced IL-25 and TSLP expression in bronchial epithelial cells via targeting the CD39–extracellular ATP axis.

We next examined whether miR-206 regulates IL-25 and TSLP expression via targeting the CD39–extracellular ATP axis in HDM-stimulated HBE cells. Inhibition of miR-206 expression by transfection with miR-206 inhibitor increased baseline and HDM-induced CD39 expression. Importantly, inhibition of miR-206 expression decreased the ATP concentration and suppressed HDM-induced IL-25 and TSLP protein expression in the medium (Supplemental Figure 3, A–D). In contrast, miR-206 overexpression by transfection with the miR-206 mimic suppressed baseline and HDM-induced CD39 expression, enhanced extracellular ATP, and further enhanced HDM-induced IL-25 and TSLP protein expression (Supplemental Figure 3, E–H). Our data indicate that miR-206 regulates epithelial IL-25 and TSLP expression by targeting the CD39–extracellular ATP axis.

### Airway epithelial miR-206 regulates CD39 expression and BALF ATP concentration in a murine model of allergic airway disease.

**Figure 5:**
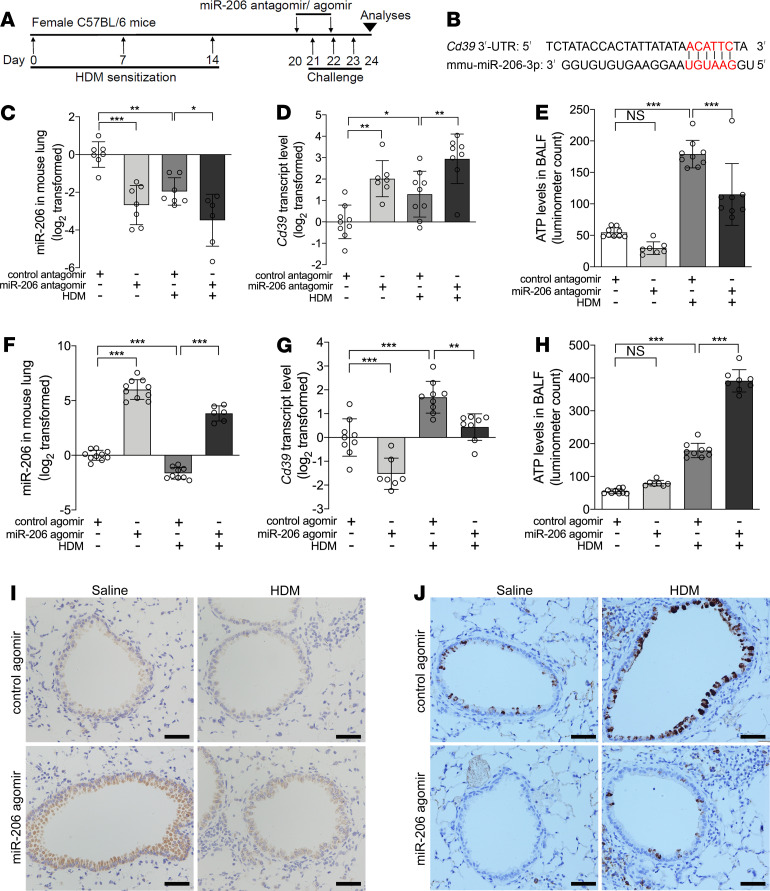
Epithelial miR-206 targets the CD39–extracellular ATP axis in a murine model of allergic airway inflammation. (**A**) Experimental schedule. (**B**) The seed region of *mmu–miR-206-3p*, and the seed recognizing sites in the 3′-UTR of mouse *Cd39* variant 1 (position 2864–2869) are shown. (**C**–**E**) miR-206 (**C**) and *Cd39* (**D**) transcript levels in the lungs and ATP levels in BALF (**E**) were determined by qPCR and luciferase bioluminescence, respectively, in mice intranasally administered control or miR-206 antagomir and challenged with HDM or saline. (**F**–**H**) miR-206 (**F**) and *Cd39* (**G**) transcript levels in the lungs and ATP levels in BALF (**H**) were determined by qPCR and luciferase bioluminescence, respectively, in mice intranasally administered control or miR-206 agomir and challenged with HDM or saline. The transcript levels are relative to the mean value of the control group and log_2_ transformed. *n* = 6–10 mice per group combined from 2 independent experiments. The data are represented as mean ± SD. *P < 0.05; **P < 0.01; ***P < 0.001 (1-way ANOVA with Bonferroni’s post hoc test). (**I** and **J**) Representative images of in situ hybridization of miR-206 (**I**) and IHC of CD39 (**J**) in lung sections from mice intranasally administered control or miR-206 agomir and challenged with HDM or saline. Scale bar: 50 μm.

We investigated the role of epithelial miR-206 in a murine model of allergic airway inflammation. C57BL/6 mice were sensitized and challenged with HDM (Figure 5A). The 3′-UTR of the mouse *Cd39* gene has binding sites for the seed region of *mmu–miR–206-3p* which is identical to *hsa–miR-206* (Figure 5B). Using qPCR, in situ hybridization, IHC, and luciferase bioluminescence, we found that HDM challenge decreased epithelial miR-206 expression, increased Cd39 expression, and markedly enhanced BALF ATP levels compared with control mice. Inhibition of airway miR-206 expression by intranasal administration of miR-206 antagomir further enhanced HDM-induced Cd39 expression but suppressed HDM-induced BALF ATP accumulation (Figure 5, C–E). In contrast, airway overexpression of miR-206 induced by intranasal administration of miR-206 agomir significantly suppressed HDM-induced Cd39 expression and further enhanced the HDM-induced increase in ATP concentrations (Figure 5, F–J). These data suggest that epithelial miR-206 targets the Cd39–extracellular ATP axis in the airway of a murine model of allergic airway disease.

### Airway miR-206 antagonism suppresses HDM-induced AHR, airway eosinophilia, mucus overproduction, and type 2 cytokine expression in mice.

**Figure 6:**
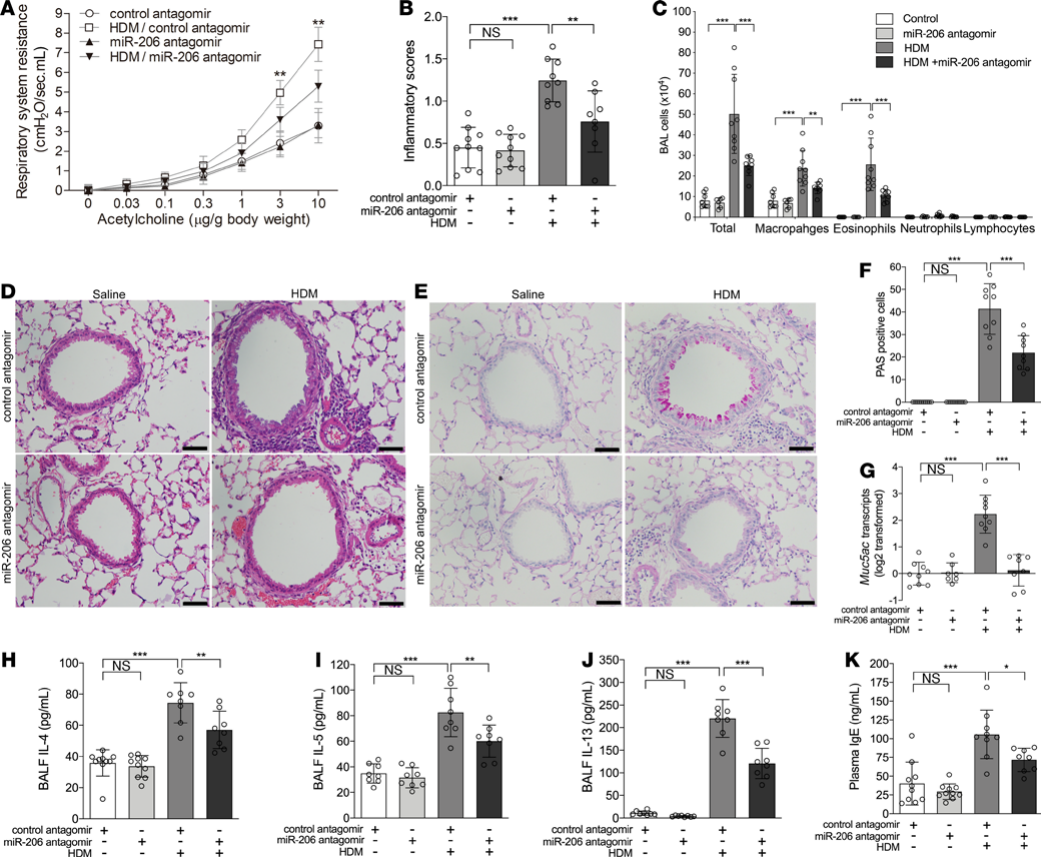
Airway miR-206 antagonism suppresses HDM-induced AHR, airway inflammation, mucus overproduction, and the type 2 response in mice. (**A**) Respiratory resistance in response to different concentrations of i.v. acetylcholine at 24 hours after the last HDM or saline challenge in mice intranasally administered with control or miR-206 antagomir. (**B**) Inflammatory scores of lung sections from mice intranasally administered with control or miR-206 antagomir and challenged with HDM or saline were calculated as described in Methods. (**C**) Counts of macrophages, eosinophils, lymphocytes, and neutrophils in BALF. (**D**) H&E staining of representative lung sections. (**E**) PAS staining for mucus in representative lung sections. (**F**) The number of PAS^+^ cells was counted in 4 random fields for each lung section at ×200 magnification. (**G**) *Muc5ac* transcript levels in mice lung were determined by qPCR. The transcript levels are relative to the mean value of the control group and log_2_ transformed. (**H**–**J**) The protein levels of IL-4 (**H**), IL-5 (**I**), and IL-13 (**J**) in BALF were determined using ELISAs. (**K**) Plasma IgE levels in peripheral blood were determined using ELISAs. *n* = 6–10 mice per group combined from 2 independent experiments. The data are represented as mean ± SD. *P < 0.05; **P < 0.01; ***P < 0.001 (1-way ANOVA with Bonferroni’s post hoc test). Scale bar: 50 μm.

In the murine model of allergic airway disease, HDM sensitization and challenge increased airway resistance to methacholine, induced infiltration of inflammatory cells around airways, and increased airway mucus-producing cells, *Muc5ac* expression, and plasma IgE levels. Inhibition of airway miR-206 expression by transfection with miR-206 antagomir significantly suppressed HDM-induced AHR, airway eosinophilic inflammation, and mucus overproduction (Figure 6, A–G). In contrast, airway miR-206 overexpression induced by transfection with miR-206 agomir further enhanced HDM-induced AHR, airway eosinophilia, and mucus overproduction (Supplemental Figure 4, A–G). Moreover, airway miR-206 antagonism suppressed HDM-induced expression of type 2 cytokines, including IL-4, IL-5, and IL-13, and decreased plasma IgE levels (Figure 6, H–K), whereas miR-206 overexpression further enhanced HDM-induced expression of type 2 cytokines in mouse lungs and plasma IgE levels (Supplemental Figure 4, H–K). Our data indicate that epithelial miR-206 plays an essential role in allergic airway disease by regulating the type 2 immune response.

### Airway miR-206 antagonism suppresses IL-25, IL-33, and Tslp expression and ILC2 expansion in mice.

**Figure 7:**
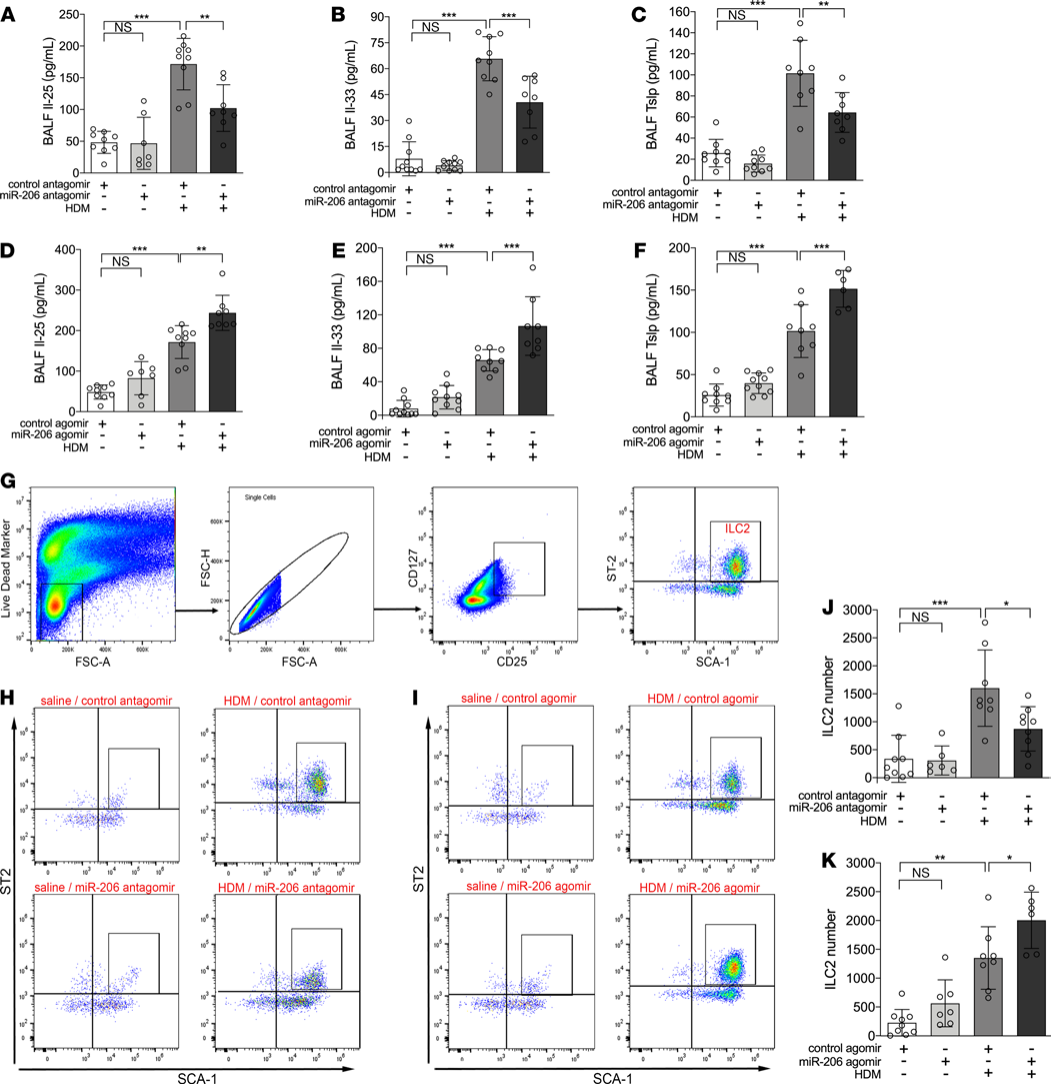
Perturbation of airway miR-206 expression alters HDM-induced IL-25, IL-33, Tslp expression and ILC2 expansion in mouse lung. (**A**–**C**) IL-25 (**A**), IL-33 (**B**), and Tslp (**C**) protein levels in BALF were determined using ELISAs in mice intranasally administered with control or miR-206 antagomir and challenged with HDM or saline. (**D**–**F**) IL-25 (**D**), IL-33 (**E**), and Tslp vprotein levels in BALF were determined using ELISAs in mice intranasally administered control or miR-206 agomir and challenged with HDM or saline. *n* = 6–10 mice per group combined from 2 independent experiments. (**G**) Single-cell suspensions of mouse lung tissue were incubated with a cocktail of biotin-conjugated antibodies for detection of lineage markers and mixed with anti-Biotin microbeads to isolate lineage^–^ lung cells. ILC2s in mouse lungs were enumerated via flow cytometry analysis with lineage^–^ lung cells using the following gating strategy: live, single, CD25^+^CD127^+^ST2^+^Sca-1^+^ cells. (**H** and **J**) Representative flow cytometric plots (**H**) and numbers of ILC2s (**J**) in the lungs of mice intranasally administered control or miR-206 antagomir and challenged with HDM or saline. (**I** and **K**) Representative flow cytometric plots (**I**) and numbers of ILC2s (**K**) in the lungs of mice intranasally administered control or miR-206 agomir and challenged with HDM or saline. *n* = 6–9 per group, combined from 2 experiments. The data are represented as mean ± SD. *P < 0.05; **P < 0.01; ***P < 0.001 (1-way ANOVA with Bonferroni’s post hoc test).

Since ILC2s play pivotal roles in the type 2 response upon activation by IL-25, IL-33, and TSLP, we further examined the effect of airway miR-206 manipulation on IL-25, IL-33, and Tslp expression and ILC2 expansion in mice. HDM sensitization and challenge increased IL-25, IL-33, and Tslp protein levels in BALF (Figure 7, A–F). Meanwhile, flow cytometric analysis revealed that HDM sensitization and challenge significantly increased the number of ILC2s (Lin^–^CD25^+^CD127^+^ST2^+^Sca-1^+^ cells, gating strategy in Figure 7G) in single-cell suspensions of lungs (Figure 7, H–K). Airway miR-206 antagonism suppressed HDM-induced IL-25, IL-33, and Tslp expression (Figure 7, A–C) and ILC2 expansion (Figure 7, H and J), whereas miR-206 overexpression further enhanced HDM-induced IL-25, IL-33, and Tslp expression (Figure 7, D–F) and ILC2 expansion in mouse lungs (Figure 7, I and K). This suggests that epithelial miR-206 regulates HDM-induced IL-25, IL-33, and Tslp expression and ILC2 expansion in mouse models.

## Discussion

In the present study, we reported that epithelial miR-206 was differentially expressed between type 2–low and –high asthma patients who were symptomatic and treatment naive. Type 2–high asthma patients had higher miR-206 expression, lower epithelial CD39 expression, elevated BALF ATP levels, and higher epithelial IL-25 and TSLP expression than type 2–low asthma patients. Of note, BALF ATP levels were strongly correlated with airway IL-25 and TSLP expression in asthma patients. The associations between these measurements were functionally validated in primary cultures of HBE cells and in a murine model of allergic airway inflammation.

miRNAs play essential roles in the pathogenesis of asthma (39, 40, 50, 51). To date, the differentially expressed epithelial miRNAs between type 2–low and type 2–high asthma remain unknown. We identified miR-206 as the most highly expressed miRNA in type 2–high asthma relative to type 2–low asthma. However, compared with control subjects, miR-206 expression was downregulated in both asthma subsets. miR-206 expression was also decreased in cultured HBE cells exposed to HDM and in the airways of mice sensitized and challenged with HDM. Our findings are consistent with a study reporting that miR-206 expression was decreased in the airway wall of a mouse model of childhood allergic asthma (52). Recent studies have shown that miR-206 expression is also decreased in mouse models of occupational asthma (53, 54). In humans, it was reported that circulating miR-206 was useful to predict childhood asthma exacerbation (55), and plasma miR-206 expression differed between asthmatics with higher and lower blood eosinophil counts (56). However, the mechanism underlying the lower reduction in epithelial miR-206 in type 2–high asthma than in type 2–low asthma requires further investigation. It was reported that production of reactive oxygen species (ROS) upregulated miR-206 expression (57, 58). Interestingly, IL-13, a type 2 cytokine, promotes ROS production in airway epithelial cells (59). Thus, one possibility for higher miR-206 expression in type 2–high asthma is that type 2 cytokine–induced ROS production upregulated miR-206 expression.

To explore the role of miR-206 in asthma, we verified that CD39, an ectonucleotidase that degrades ATP, was a target of miR-206. To date, there are no studies addressing the regulation of airway CD39 in human asthma, to our knowledge. Previous reports regarding the role of Cd39 in animal asthma models are conflicting (32, 33). We demonstrate that CD39 expression was increased in the airway epithelium in human asthma and in mice sensitized and challenged with HDM. Consistent with the higher expression of miR-206 in type 2–high asthma, CD39 expression was lower in type 2–high asthma than in type 2–low asthma.

Extracellular ATP serves as a danger signal to alert the immune system of damaged tissue. In our cohort of symptomatic and treatment-naive asthma patients, BALF ATP concentrations were significantly increased, especially in type 2–high asthma patients. This is consistent with a previous report that allergen provocation enhanced airway BALF ATP accumulation in asthma patients (28). The elevated airway ATP levels in our asthma patients might be due to exposure to environmental aeroallergens, including HDM and/or airway virus infections, the main triggers of asthma exacerbation (12, 60). Rhinovirus infection has been reported to stimulate bronchial smooth muscle cells to release ATP (61), and influenza A virus infection was found to increase BALF ATP levels in mice (62).

In our in vitro system, we demonstrate that allergen-induced acute extracellular ATP accumulation was responsible for miR-206 downregulation and CD39 upregulation in HBE cells at an air-liquid interface. Since CD39 catalyzes ATP degradation, the CD39 upregulation in asthma patients and in allergen-exposed epithelial cells may represent an inhibitory feedback response to excessive ATP, through which the maintenance of airway homeostasis may be possible.

In human asthma, we previously reported that epithelial *IL25* mRNA expression was upregulated in a subset of asthma patients featured by type 2 inflammation (63). Here, in a different cohort of asthmatic patients, using ELISA and qPCR, we found that airway expression of IL-25 and TSLP, but not IL-33, was elevated in type 2–high asthma patients. The TaqMan qPCR primers and probes for the *IL33* isoform without exons 3 and 4, and the long isoform of *TSLP* were previously reported, and these isoforms were associated with type 2 inflammation (48, 49). To date, there have been few head-to-head studies of IL-25, IL-33, and TSLP expression in human asthma. Various expression patterns of these cytokines have been reported in different populations. In Korea, plasma IL-25, but not IL-33 or TSLP, was increased in patients with aspirin-exacerbated respiratory disease characterized by asthma, nasal polyps, and chronic eosinophilic sinusitis (21). Elevated IL-25, but not IL-33 or TSLP mRNA, was reported in sputum cells from uncontrolled asthmatics in Belgium (22). In contrast, increased expression of IL-33 and TSLP, but not IL-25, was reported in asthma patients in London (23), Poland (24), and New York, New York (25). This suggests that ethnicity and region should be considered in studies on these cytokines. Recently, a new concept — regiotype — was introduced for allergic diseases, referring to regional differences between endotypes due to different allergens and other environmental influences (2). In chronic rhinosinusitis with nasal polyps (CRSwNP), a disease related to asthma, various expression patterns of IL-25, IL-33, and TSLP in different populations and regions have been described. Reports of IL-25 upregulation in CRSwNP have come from Asian countries including Korea (64), Japan (65), and China (66), whereas negative results for IL-25 were reported from the USA (67), Australia (68), and Turkey (69). Our data on airway IL-25, IL-33, and TSLP expression in asthma patients provide evidence for the potentially novel therapies targeting these cytokines in China.

The upstream signaling pathway regulating the expression of epithelial IL-25 and TSLP remains largely unknown. Extracellular ATP–activated purinergic receptors mediate aeroallergen-induced IL-33 release from epithelial cells (34). Here, we report that BALF ATP concentrations were strongly correlated with airway IL-25 and TSLP expression. In vitro, allergen-induced acute accumulation of extracellular ATP was required and sufficient for IL-25 and TSLP expression. Furthermore, epithelial miR-206 regulated IL-25 and TSLP expression by targeting the CD39–extracellular ATP axis both in vitro and in vivo. HDM sensitization and challenge decreased airway miR-206 expression, while increasing Cd39 expression; BALF ATP concentration; IL-25, IL-33, and Tslp expression; and ILC2 expansion in mice. Airway miR-206 antagonism before HDM challenge suppressed IL-25, IL-33, and Tslp expression; ILC2 expansion; type 2 cytokine expression; and the cardinal features of asthma in mice. On the other hand, miR-206 overexpression had the opposite effects. The explanation of the effect of miR-206 agomir transfection should be interpreted with caution because nonspecific effect of miRNA overexpression was reported (70). In addition, intranasal administration of miR-206 agomir or antagomir may also affect Cd39 expression in other cell types, including macrophages in mice lungs.

**Figure 8:**
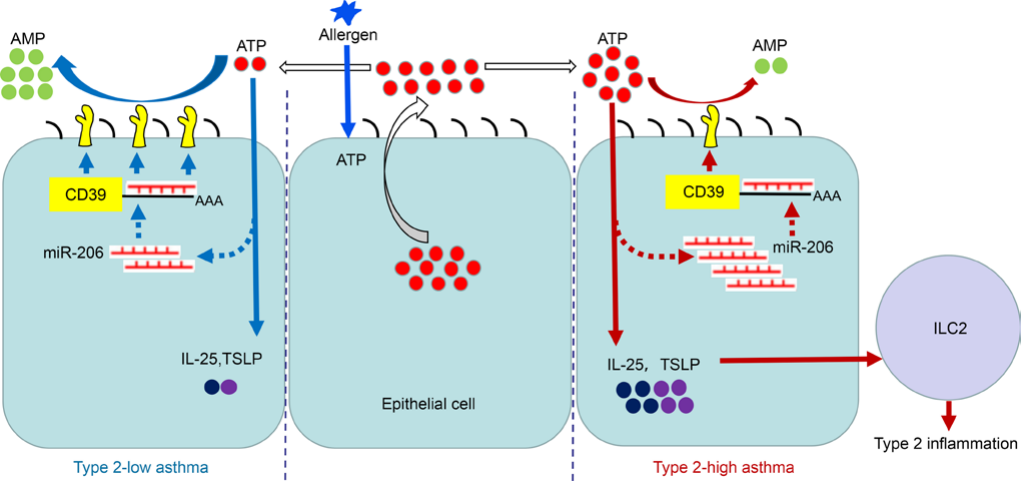
Scheme illustrating for the signaling pathway regulating IL-25 and TSLP expression in airway epithelial cells in type 2–low and type 2–high asthma. Allergens stimulate rapid release of ATP from epithelial cells. Extracellular ATP serves as an alarmin to induce expression of the innate cytokines IL-25 and TSLP. Meanwhile, acute accumulation of extracellular ATP decreases epithelial miR-206 expression, which upregulates CD39 expression to eliminate excessive ATP. Epithelial miR-206 is decreased in both type 2–low and type 2–high asthma. Compared with type 2–low asthma, less reduction in epithelial miR-206 results in higher miR-206 level, lower CD39 expression, and impaired capacity to eliminate extracellular ATP in type 2–high asthma. Consequently, more extracellular ATP accumulates, which leads to higher expression of IL-25 and TSLP and more prominent type 2 inflammation in type 2–high asthma.

As summarized in Figure 8, our findings suggest that epithelial miR-206 is downregulated in both asthma subsets. Compared with type 2–low asthma, higher miR-206 expression resulted in lower CD39 expression and impaired capacity to eliminate extracellular ATP in type 2–high asthma. Consequently, more extracellular ATP was accumulated, leading to higher expression of IL-25 and TSLP and more prominent type 2 inflammation in type 2–high asthma. The mechanism underlying the impaired ability to downregulate miR-206 in response to excessive ATP in type 2–high asthma requires further study.

In conclusion, epithelial miR-206 regulates airway IL-25 and TSLP expression and type 2 inflammation in asthma by targeting the CD39–extracellular ATP axis. This pathway contributes, at least in part, to the development of human type 2–high asthma and represents a potentially novel therapeutic target for this endotype.

## Methods

### Human subjects.

We recruited 26 healthy control subjects and 57 asthma patients who were symptomatic, newly diagnosed, and treatment naive. All subjects were Chinese and were recruited from Tongji Hospital. Subjects with asthma were diagnosed by a physician; had symptoms of episodic cough, wheeze, and/or dyspnea; and had an accumulated dosage of methacholine provoking a 20% fall (PD_20_) in FEV_1_ < 2.505 mg and/or ≥ 12% increase in FEV_1_ following inhalation of 200 μg salbutamol. We recruited subjects who had never smoked and had never received inhaled or oral corticosteroids or leukotriene antagonists. Healthy control subjects had no respiratory symptoms, normal spirometric values, and a methacholine PD_20_ ≥ 2.505 mg.

We recorded demographic information, performed spirometry, measured FeNO, and collected induced sputum. We performed bronchoscopy with BAL and bronchial brushing. After inspection of the bronchial tree, 40 mL of prewarmed 0.9% saline was instilled into the right middle lobe and then gently aspirated. We brushed 10 sites within the subsegmental bronchi of the right middle and lower lobes (10 gentle upward and downward strokes per site). The dissociated cells were recovered in ice-cold DMEM.

### MicroRNA microarray.

Total RNA from bronchial epithelial brushing samples from 4 type 2–low and 4 type 2–high asthma patients was extracted using TRIzol (Invitrogen). After RNA quantity measurement using a NanoDrop 1000 spectrophotometer, the samples were labeled using a miRCURY Hy3/Hy5 power labeling kit (Exiqon) and hybridized on a miRCURY LNA microRNA array (seventh generation, miRBase v18; Exiqon). The slides were scanned using an Axon GenePix 4000B microarray scanner (Axon Instruments). Scanned images were then imported into GenePix Pro 6.0 software (Axon Instruments) for grid alignment and data extraction. We used the median normalization method to obtain normalized data, which can be defined with the following equation: normalized data = (foreground – background)/median. In comparison, genes with greater than 2-fold change and that showed a statistically significant difference between the 2 groups were considered to be differentially expressed. The data are deposited at GEO (http://www.ncbi.nlm.nih.gov/geo/, accession no. GSE142237).

### Cell culture and treatment.

HBE cells collected from healthy donors (*n* = 8) using the bronchial brushing technique were cultured on an air-liquid interface as previously described (71, 72). Briefly, 10 sites of the subsegmental bronchi of the right middle and lower lobes were brushed. The dissociated cells were recovered by vortexing the brush in ice-cold DMEM. The cells were centrifuged, resuspended, seeded into 6-well plates coated with collagen I from rat tails (Corning), and grown in bronchial epithelial cell medium (BEpiCM; ScienCell) with supplements. The medium was changed every 48 hours until the cells were 90% confluent. Cells were then seeded on 1.1 cm^2^ Transwell inserts (Corning) with 0.4 μm pores. Cells were submerged for the first 7 days in BEpiCM (ScienCell) with bronchial epithelial cell growth supplement (ScienCell) and penicillin/streptomycin solution, and then the apical medium was removed to establish an air-liquid interface that was maintained for the next 14 days. The basolateral medium was changed to differentiation medium containing a 1:1 mixture of DMEM (HyClone) and bronchial epithelial cell growth medium (BEGM; Lonza) with supplements and 50 nM all-trans retinoic acid (Sigma-Aldrich). Cells were stimulated with HDM (50 μg/mL; Greer Laboratories) and transfected with control or miR-206 mimic, control or miR-206 inhibitor (RiboBio), scrambled or CD39 siRNA, and an empty or CD39 cDNA expression vector. Cells were also stimulated with HDM with or without apyrase or ATPγS (Sigma-Aldrich). BEAS-2B cell lines were purchased from ATCC. Cells were cultured in DMEM medium with 10% FBS and stimulated with *Alternaria* (50 μg/mL; Greer Laboratories) with or without apyrase.

### Mouse model of allergic airway inflammation.

Female C57BL/6 mice were obtained from the Experimental Animal Center of Hubei Province (Wuhan, China). The model was established by HDM sensitization and challenge. Briefly, female C57BL/6 mice received an i.p. injection of 100 μL of a solution of lyophilized HDM extract (0.1 mg/mL; Greer Laboratories) and Al(OH)_3_ as an adjuvant on days 0, 7, and 14, and received 40 μL of HDM solution (3 mg/mL) or saline intranasally on days 21, 22, and 23. miR-206 agomir (5 nmol in 40 μL saline; RiboBio), control agomir, miR-206 antagomir (20 nmol in 40 μL saline), or control antagomir were administered intranasally on days 20 and 22. Twenty-four hours after the last HDM challenge, respiratory resistance in response to a range of concentrations of i.v. acetylcholine was measured using the forced oscillation technique with the FlexiVent system (SCIREQ) as previously described (73).

### Assessment of mouse airway inflammation.

Cell counts for macrophages, eosinophils, lymphocytes, and neutrophils in BALF were performed as previously described (73). Paraffin-embedded 5 μm lung sections were stained with H&E. The severity of peribronchial inflammation was scored by a blinded observer using the following features: 0, normal; 1, few cells; 2, a ring of inflammatory cells 1 cell layer deep; 3, a ring of inflammatory cells 2–4 cells deep; 4, a ring of inflammatory cells > 4 cells deep.

### IHC.

Sections of mouse lungs were stained with rabbit polyclonal CD39 (ENTPD1) antibody (Proteintech). Antibodies were detected using the Real EnVision detection system (Dako Diagnostics) according to the instructions.

### PAS staining.

Mouse lung sections were stained with periodic acid–Schiff (PAS) (Goodbio Technology) for detection of mucus. The number of PAS^+^ cells was counted in 4 random fields for each lung section at ×200 magnification.

### qPCR.

Total RNA was isolated and reverse-transcribed to quantify hsa–miR-206 expression in epithelial brushings and HBE cells; mmu–miR-206 expression in mouse lungs; *CD39*, *IL-25*, *IL-33* (transcript without exons 3 and 4), *TSLP* (long isoform), *CLCA1*, *POSTN*, and *SERPINB2* mRNA expression in epithelial brushings; and *Cd39* mRNA expression in mouse lungs. qPCR was performed using an ABI Prism 7500 HT Fast Real-time PCR System (Applied Biosystems). The Ct of each gene transcript was normalized to the Ct of U6 or U48 for miRNA and to β-actin or *GAPDH* for mRNA. Relative gene expression was calculated using the 2^–ΔΔCt^ method (74). The transcript levels of each gene are expressed as relative to the median level in healthy control subjects or the mean of the control group and log_2_ transformed. Primers for qPCR are listed in Supplemental Table 1. The stem-loop RT primer (ssD809230234), forward primer (ssD809230926), and reverse primer (ssD089261711) for hsa–/mmu–miR-206 were from RiboBio. TaqMan primer and probe sets for *IL25* (Hs00224471_m1), *TSLP* (long isoform; Hs01572933_m1), and *ACTB* (Hs99999903_m1) were obtained from Applied Biosystems. The transcripts for *IL33* without exons 3 and 4 were determined by RNase H–dependent qPCR as reported by Gordon et al. (48).

### In situ hybridization.

We performed in situ hybridization of mmu–miR-206 on paraffin-embedded sections using mmu–miR-206 miRCURY LNA miRNA detection probe (Qiagen). The sequence of the probes for mmu–miR-206 was 5′-CCACACACTTCCTTACATTCCA-3′.

### Luciferase activity assay.

BEAS-2B cells were cotransfected with vector harboring the WT, mutant *CD39* 3′-UTR or no 3′-UTR (control) and with miR-206 mimic or nontargeting control. Luciferase activity was detected with a dual luciferase reporter assay system (Promega). Normalized RLUs represent firefly luciferase activity/Renilla luciferase activity.

### Western blotting.

CD39 protein expression in cells was detected with monoclonal mouse anti–human CD39 antibody (clone OTI2B10, OriGene Technologies) using Western blotting as previously described (73).

### ELISA.

Human IL-25 (RayBiotech), IL-33, and TSLP (R&D Systems) in supernatant from BALF and cell culture medium, and mouse IL-4, IL-5, IL-13 (R&D Systems), IL-25, IL-33 (Thermo Fisher Scientific), and Tslp (R&D Systems) in BALF supernatant were measured via ELISA according to manufacturer instructions. Mouse plasma IgE levels were determined via ELISA (Dakewe Biotech). All samples and standards were measured in duplicate.

### ATP measurements.

To measure ATP levels in human BALF, ice-cold BALF samples were centrifuged at 300*g* at 4°C immediately after collection, and the supernatants were stored at –80°C. Supernatants of fresh mouse BALF and cell culture medium were analyzed immediately. ATP levels were measured using an ATP assay kit (Beyotime Biotechnology) according to the instructions.

### Flow cytometry.

To analyze lung ILC2s, single-cell suspensions of mouse lung tissue were incubated with a cocktail of biotin-conjugated monoclonal antibodies to detect lineage markers (CD5, CD11b, CD45R [B220], anti–Gr-1 [Ly-6G/C], 7-4, and Ter-119) and then mixed with anti-Biotin MicroBeads (Miltenyi Biotec). Lineage^–^ lung cells were isolated with a column placed in the magnetic field of a MACS Separator (Miltenyi Biotec). Lineage^–^ cells were stained with BV421-conjugated Live/Dead Fixable Dead Cell Stain (Invitrogen), PerCP/Cy5.5-conjugated CD25 (clone PC61; BioLegend), PE-conjugated CD127 (clone A7R34; BioLegend), FITC-conjugated T1/ST2 (clone DJ8; MD bioscience), and APC-conjugated Sca-1 (clone D7; BioLegend) antibodies. The samples were analyzed using an EPICS-XL MCL flow cytometer (Beckman Coulter). Live lineage^–^ CD25^+^CD127^+^T1/ST2^+^Sca-1^+^ lymphocytes were identified as ILC2s. Data were analyzed with FlowJo software (TreeStar).

### Statistics.

We analyzed data using Prism version 5 (GraphPad Software) and SPSS version 19 (SPSS Inc.). For normally distributed data, we calculated the means ± SD and used parametric tests (unpaired Student’s *t* test or 1-way ANOVA with Bonferroni’s post hoc test). For nonnormally distributed data, we calculated the medians (with IQRs) and used nonparametric tests (Mann-Whitney *U* test or 1-way ANOVA with Bonferroni’s post hoc test). We analyzed correlations using Spearman’s rank order correlation. Values of *P* < 0.05 were considered statistically significant.

### Study approval.

Human and mouse studies were approved by the ethics committee of Tongji Hospital, Tongji Medical College, Huazhong University of Science and Technology. Participants gave informed consent.

## Author contributions

GZ designed the research, conceived of the manuscript, and had the primary responsibility for writing. KZ, YF, YL, WW, CC, D Chen, SC, JG, and GC performed experiments. KZ, YF, YL, LY, D Cheng, and GZ analyzed data. KZ, YF, YL, LY, D Cheng, and GZ interpreted results of experiments. KZ, YF, and GZ prepared figures and drafted the manuscript. GZ edited and revised the manuscript. All authors reviewed and approved the manuscript.

## Supplementary Material

Supplemental data

## Figures and Tables

**Table 1:**
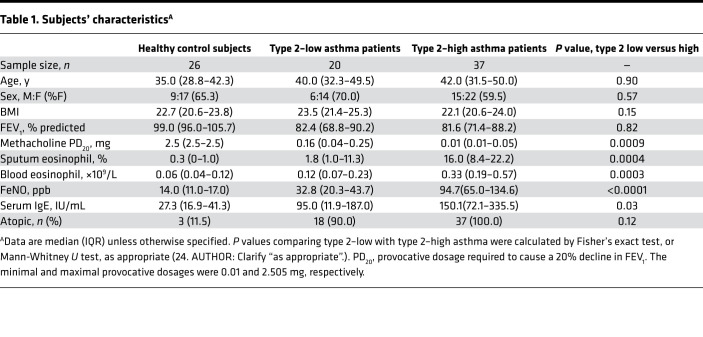
Subjects’ characteristics^A^

## Version 1 05/04/2021

In-Press Preview

## Version 2 06/08/2021

Electronic publication
