# Supplementary material for: Epithelial miR-206 targets CD39/extracellular ATP to upregulate airway IL-25 and TSLP in type 2–high asthma
Source: JCI Insight. 2021 Jun 8;6(11):e148103. doi: 10.1172/jci.insight.148103 (PMC8262281; doi:10.1172/jci.insight.148103)
Supplement: Supplemental data [file jciinsight-6-148103-s177.pdf]

Online Data Supplement:

Epithelial microRNA-206 targets CD39/extracellular ATP to upregulate airway IL25 and TSLP in type 2-high asthma

Kan Zhang[1,2,3]*, Yuchen Feng[1,2]*, Yuxia Liang[1,2], Wenliang Wu[1,2], Chenli Chang[1,2], Dian Chen[1,2], Shengchong Chen[1,2], Jiali Gao[1,2], Gongqi Chen[1,2], Lingling Yi[1,2], Dan Cheng[4], Guohua Zhen[1,2]†

[1]Division of Pulmonary and Critical Care Medicine, Department of Internal Medicine, Tongji Hospital, Tongji Medical College, Huazhong University of Science and Technology, Wuhan, China; [2]Key Laboratory of Respiratory Diseases, National Health Commission of People's Republic of China, and National Clinical Research Center for Respiratory Diseases, Wuhan, China; [3]Department of Respiratory and Critical Care Medicine, The Affiliated Suzhou Hospital of Nanjing Medical University, Suzhou, China; [4]Department of Respiratory and Critical Care Medicine, Renmin Hospital of Wuhan University, Wuhan, China.

Supplementary Table. Primers for quantitative PCR

Gene	Species	Type	Sequence
β-actin	Human	Forward	GCAAGCAGGACTATGACGAG
		Reverse	CAAATAAAGCCATGCCAATC
CD39	Human	Forward	ACTATCGAGTCCCCAGATAATGC
		Reverse	CCTGATCCTTCCCATAGCACAA
CLCA1	Human	Forward	ATGGCTATGAAGGCATTGTCG
		Reverse	TGGCACATTGGGGTCGATTG
GAPDH	Human	Forward	AAGGTGAAGGTCGGAGTCAAC
		Reverse	GGGGTCATTGATGGCAACAATA
POSTN	Human	Forward	GACCGTGTGCTTACACAAATTG
		Reverse	AAGTGACCGTCTCTTCCAAGG
SERPINB2	Human	Forward	TCCTGGGTCAAGACTCAAACC
		Reverse	CATCCTGGTATCCCCATCTACA
β-actin	Mouse	Forward	GGCTGTATTCCCCTCCATCG
		Reverse	CCAGTTGGTAACAATGCCATGT
Gapdh	Mouse	Forward	TGGCCTTCCGTGTTCCTAC
		Reverse	GAGTTGCTGTTGAAGTCGCA
Cd39	Mouse	Forward	AGATGAAATCGGTGCGTACCT
		Reverse	GAGTCTGGTGATGCTTGGATG

Supplementary Figures and Figure Legends:

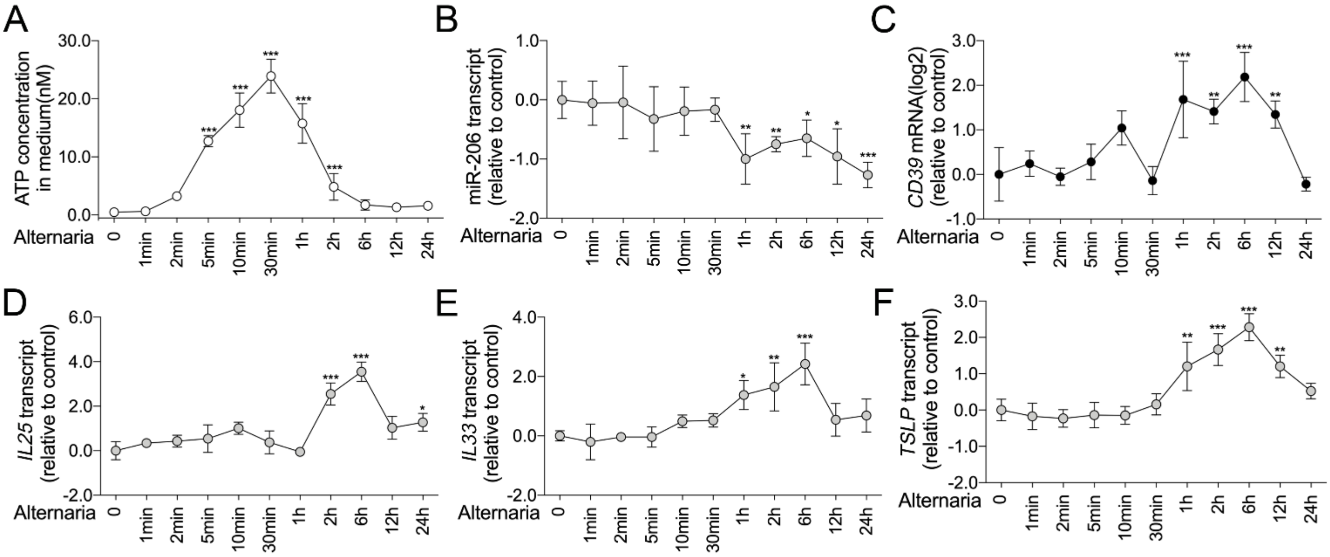

Supplementary Figure 1. The kinetics of extracellular ATP and the expression of miR-206, *CD39*, *IL25*, *IL33*, and *TSLP* in BEAS-2B cells after Alternaria stimulation. (A) ATP concentration in BEAS-2B cells culture medium collected at indicated time points after Alternaria (50 μg/ml) stimulation was measured by luciferase bioluminescence. (B-F) Transcript levels of miR-206 (*B*), *CD39* (*C*), *IL25* (*D*), *IL33* (*E*) and *TSLP* (*F*) in BEAS-2B cells harvested at indicated time points after Alternaria (50 μg/ml) stimulation were determined by quantitative PCR. n = 4 wells per group. Data are mean ± SD. **P* < 0.05; ***P* < 0.01; ****P* < 0.001; *****P* <0.0001 (one-way ANOVA with Bonferroni's post hoc test).

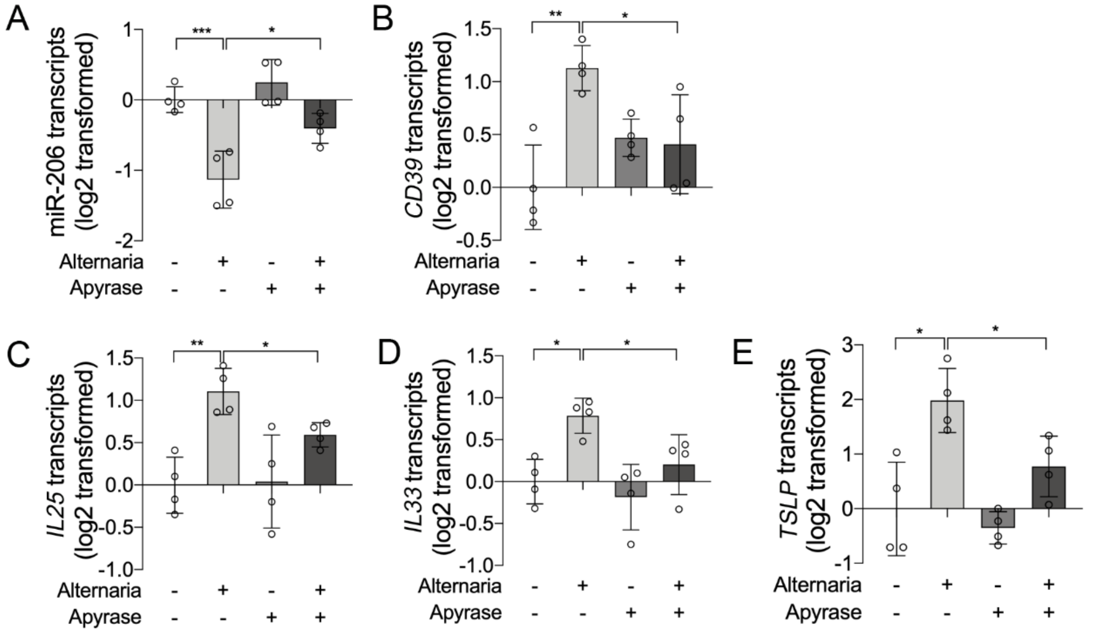

Supplementary Figure 2. Extracellular ATP is required for Alternaria-induced miR-206, *CD39*, *IL25*, *IL33* and *TSLP* expression in BEAS-2B cells. (A-B) miR-206 (*A*) and *CD39* (*B*) transcript levels in BEAS-2B cells pretreated with apyrase or saline for 2 h before adding Alternaria (50 µg/ml) and stimulation for 6 h. The transcript levels are expressed as relative to the mean value of control group and log2 transformed. (C-D) *IL25* (*C*), *IL33* (*D*) and *TSLP* (*E*) transcript levels in BEAS-2B cells pretreated with apyrase or saline for 2 h before adding Alternaria (50 µg/ml) and stimulation for 6 h. The transcript levels are expressed as relative to the mean value of control group and log2 transformed. n = 4 wells per group. The data are shown as mean ± SD. *$P < 0.05$; **$P < 0.01$; ***$P < 0.001$ (one-way ANOVA with Bonferroni's post hoc test).

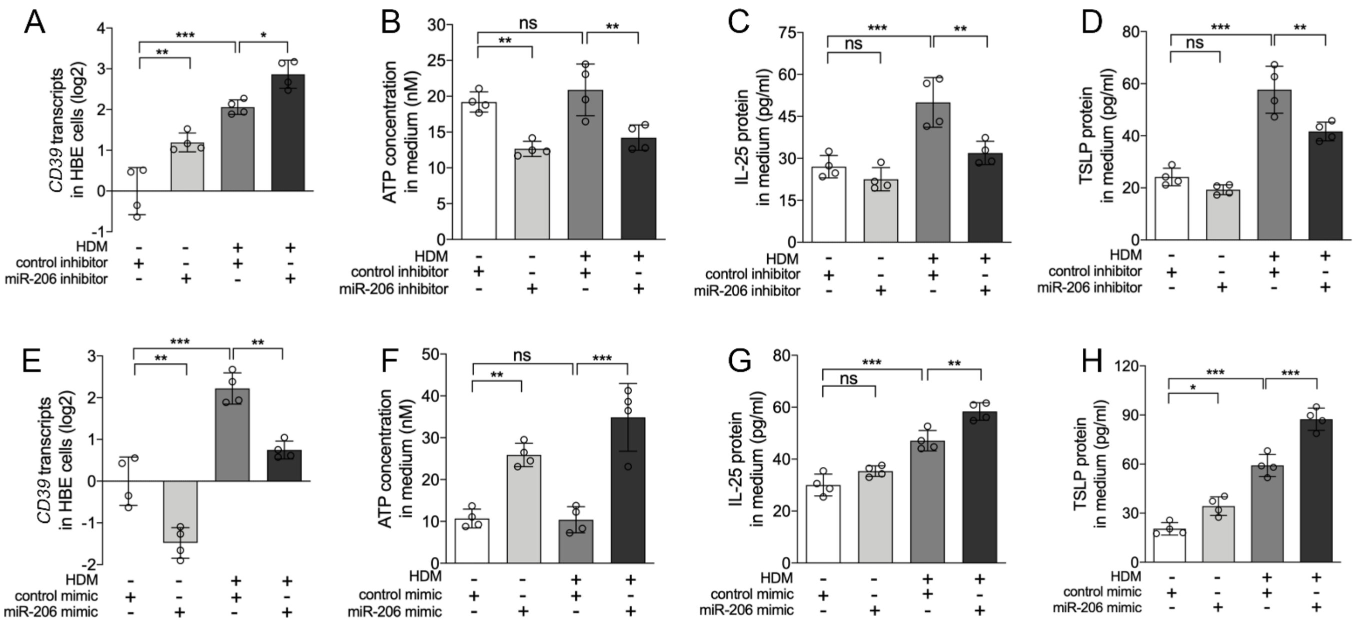

Supplementary Figure 3. MiR-206 regulates allergen-induced IL-25 and TSLP expression via targeting CD39 - extracellular ATP axis in bronchial epithelial cells. (A) *CD39* transcript levels in HBE cells transfected with control or miR-206 inhibitor and stimulated with or without HDM for 6 h were determined by quantitative PCR. (B) ATP concentration in culture medium after transfection with control or miR-206 inhibitor and stimulation with or without HDM for 6 h were measured by luciferase bioluminescence. (C, D) IL-25 (*C*), and TSLP (*D*) protein levels in culture medium after transfection with control or miR-206 inhibitor and stimulation with or without HDM for 6 h were determined by ELISA. (E) *CD39* transcript levels in HBE cells transfected with control or miR-206 mimic and stimulated with or without HDM for 6 h were determined by quantitative PCR. (F) ATP concentration in culture medium after transfection with control or miR-206 mimic and stimulation with or without HDM for 6 h were measured by luciferase bioluminescence. (G, H) IL-25 (*G*), and TSLP (*H*) protein levels in culture medium after transfection with control or miR-206 mimic and stimulation with or without HDM for 6 h were determined by ELISA. n = 4 wells per

group combined from 2 experiments using HBE cells from 2 healthy donors. Data are mean ± SD. $*P < 0.05$; $**P < 0.01$; $***P < 0.001$ (one-way ANOVA with Bonferroni's post hoc test).

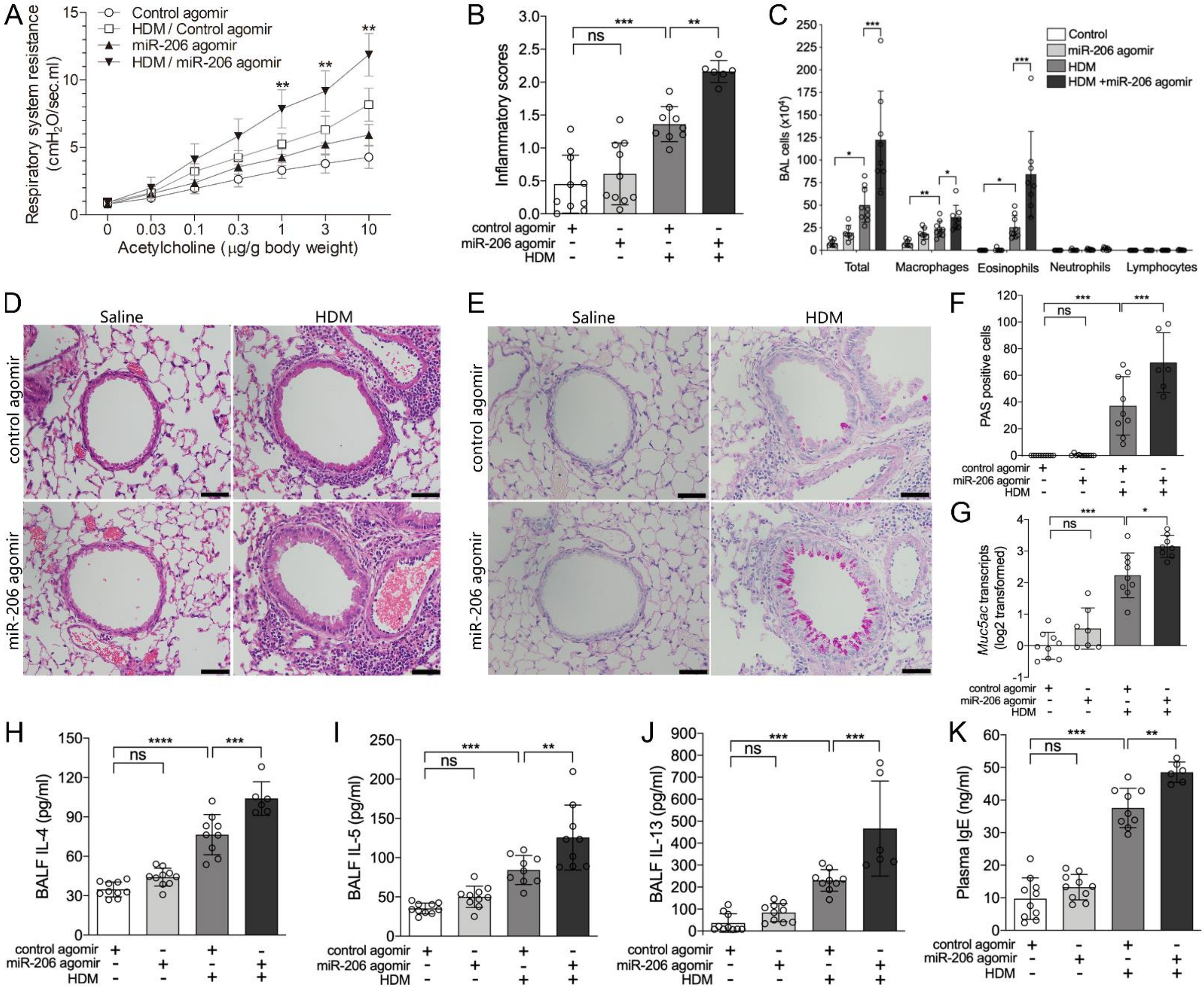

Supplementary Figure 4. Overexpression of airway miR-206 expression aggravates HDM-induced AHR, airway inflammation, mucus overproduction and type 2 response in mice. (A) Respiratory resistance in response to different concentration of intravenous acetylcholine at 24 h after the last HDM or saline challenge in mice intranasally administered with control or miR-206 agomir. (B) Inflammatory scores of lung sections from mice intranasally administered with control or miR-206 agomir and challenged with HDM or saline were calculated as described in

Methods. (C) Counts for macrophages, eosinophils, lymphocytes and neutrophils in BALF. (D) H&E staining of representative lung sections. (E) PAS staining for mucus in representative lung sections. (F) The numbers of PAS-staining-positive cells were counted in four random fields for each lung section at ×200 magnification. (G) *Muc5ac* transcript levels in mice lung were determined by quantitative PCR. The transcript levels are expressed as relative to the mean value of the control group and log2 transformed. (H-J) The protein levels of IL-4 (*H*), IL-5 (*I*), IL-13 (*J*) in BALF were determined by ELISA. (K) Plasma IgE levels in peripheral blood were determined by ELISA. n = 6-10 mice per group combined from 2 experiments. Data are mean ± SD. *$P < 0.05$; **$P < 0.01$; ***$P < 0.001$ (one-way ANOVA with Bonferroni's post hoc test). Scale bar = 50 μm.
